# Gasless Laparoscopic Surgery for Minimally Invasive Surgery in Low-Resource Settings: Methods for Evaluating Surgical Field of View and Abdominal Wall Lift Force

**DOI:** 10.1177/1553350620964331

**Published:** 2020-10-06

**Authors:** William S. Bolton, Noel K. Aruparayil, Manish Chauhan, William R. Kitchen, Kevin J. N. Gnanaraj, Alice M. Benton, Sophie E. Hutchinson, Joshua R. Burke, Jesudian Gnanaraj, David G. Jayne, Peter R. Culmer

**Affiliations:** 1 Leeds Institute of Medical Research at St James’s, 4468 University of Leeds, UK; 2 School of Mechanical Engineering, 4468 University of Leeds, UK; 3 School of Clinical Medicine, Cambridge Biomedical Campus, 2152 University of Cambridge, UK; 4 Department of Mechanical Engineering, 7235 University of Saskatchewan, Canada; 5 121735 Karunya Institute of Technology and Science, Coimbatore, India

Dear Editors:

Laparoscopic surgery has advantages over open surgery for several abdominal conditions due to improved short-term outcomes.^1^ Performing laparoscopic surgery in many low and middle-income country (LMIC) settings is restricted by the lack of general anaesthesia (GA) and carbon dioxide (CO_2_) insufflation. Gasless laparoscopic surgery employs the use of a mechanical anterior abdominal wall lift device to create internal space within the abdomen. This negates the need for GA and CO_2_ which may help increase adoption of laparoscopic surgery in LMIC settings.^2^

The safety and efficacy of gasless techniques appear to be non-inferior when compared to conventional laparoscopic surgery for many gastrointestinal and gynaecological conditions.^3^ However, concerns from surgeons before adopting this technique are operative field of view and safety concerns including damage to the abdominal wall during the lift.^3^ Many lift devices produce a tenting effect, creating an angular cavity that can restrict view.^3^ Monitoring to ensure a ‘safe’ force is applied is also essential, as lifting the abdominal wall carries the potential for trauma if too much force is applied.^4^ Our aim was to develop methods that may be used in future clinical studies aimed at mitigating these concerns by assessing field of view and force exerted on tissues during gasless lift procedures.

Firstly, we developed a novel mechanical force sensor and integrated this into a lift device. The sensor indicated the magnitude and estimated ‘safety’ of the vertical load applied to the wall during lifting. Using a frugal design approach, we developed a ‘spring-balance’ system which provides a robust low-cost mechanism that can be readily manufactured and maintained in LMICs (Figure 1). The gauge consists of a spring housed within 2 concentric tubes, combined with a calibrated colour scale on the outside of the inner tube to inform the user of the load being applied. The sensor was calibrated to display Green (Force = 0-130N), Yellow (Force = 130-150N), and Red (Force = 150-170N). These values align with literature values for acceptable force ranges exerted during conventional laparoscopic surgery.^5^ The sensor can be readily integrated into a low-cost lift system and would be ideal during training to provide the user with objective visual feedback on loading.

**Figure 1.**
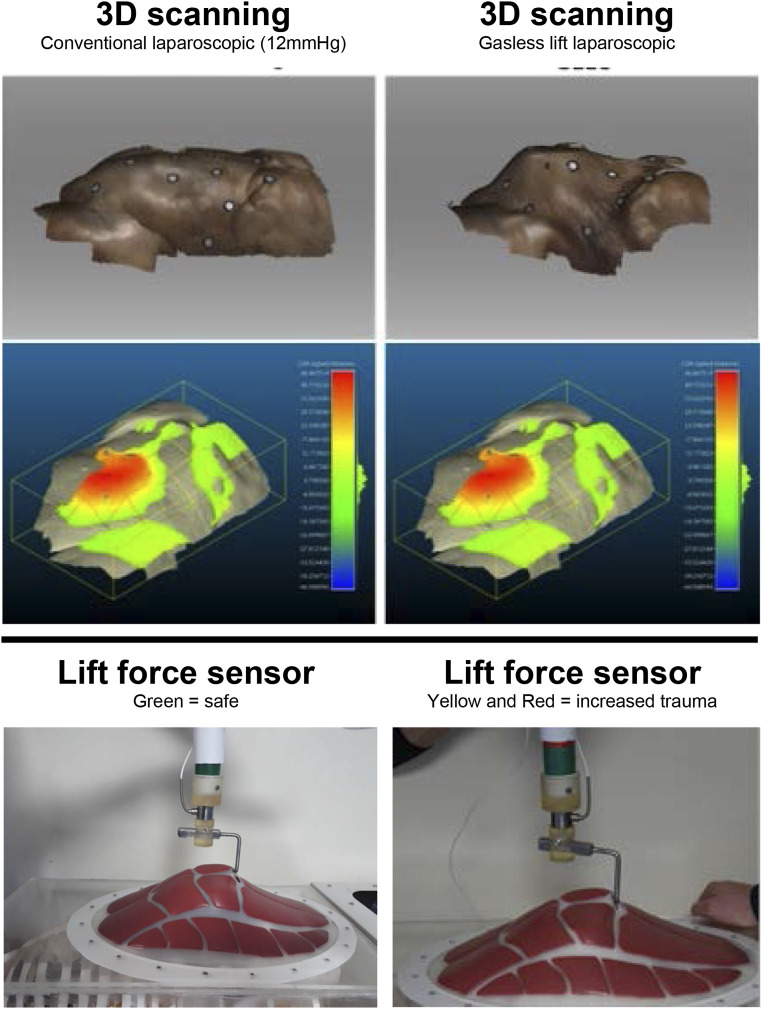
3D scanning technology and a novel mechanical force sensor can be used to evaluate field of view and safe lift forces during gasless laparoscopic experiments.

Secondly, we conducted an experiment using a single human cadaver. The purpose of this was to assess the field of view via 3 dimensional (3D) abdominal wall surface scanning and volumetric analysis. Intra-abdominal volume created via the techniques was used as the proxy for field of view. Paper reference markers were sutured to the abdominal surface to aid registration during the surface scanning that was carried out using a commercially available 3D laser scanner (Artec Space Spider, Artec 3D). The associated scans were processed using a bespoke software package (Artec Studio 12, Artec 3D) and the resultant surfaces of the cadaveric abdominal wall were analysed to obtain cross-sectional data using an open source 3D mesh analysis software (CloudCompare). The headroom profile and abdominal wall shape created by conventional laparoscopic surgery at 12 mmHg and gasless lift were compared (Figure 1). While some ‘tenting’ effects are apparent, similar volumes were achieved by gasless techniques as compared to insufflation. Further validation as to the impact of field of view in clinical studies is required.

Our results demonstrate the use of the 3D surface scanning technology and the novel mechanical force sensor are suitable methods to evaluate field of view and force applied to the abdominal wall. These methods have relevance to support further evaluation of gasless surgery techniques in clinical trials with living patients.

## Dissemination Declaration

The results will be disseminated on social media platforms and reports back to funders.

## Author Contributions

William S. Bolton, Peter R. Culmer, Jesudian Gnanaraj and David G. Jayne conceptualised the study. William S. Bolton, Noel K. Aruparayil, Joshua R. Burke and William R. Kitchen arranged the cadaveric assessments. Manish Chauhan, Kevin J. N. Gnanaraj, Alice M. Benton, and Sophie E. Hutchinson led the development of the mechanical force sensor and conducted volumetric analysis. William S. Bolton prepared the first draft of the manuscript which was subsequently edited by all authors. David G. Jayne is the study guarantor.

Study concept and design: William S. Bolton, Peter R. Culmer, Jesudian Gnanaraj, and David G. Jayne

Acquisition of data: William S. Bolton, Noel K. Aruparayil, Joshua R. Burke, and William R. Kitchen

Analysis and interpretation: Manish Chauhan, Kevin J. N. Gnanaraj, Alice M. Benton, and Sophie E. Hutchinson

Study supervision: David G. Jayne and Peter R. Culmer
